# Signal, detection and estimation using a hybrid quantum circuit

**DOI:** 10.1038/s41598-024-65520-4

**Published:** 2024-07-02

**Authors:** O. P. de Sá Neto, M. C. de Oliveira

**Affiliations:** 1 Coordenação de Ciências da Computação, Universidade Estadual do Piauí, Campus Professor Alexandre Alves de Oliveira, Parnaíba, Piauí 64202–220 Brazil; 2 Instituto de Física Gleb Wataghin, Universidade Estadual de Campinas, Campinas, São Paulo 13083-970 Brazil

**Keywords:** Hybrid circuit, IQ mix detection, Quantum switch, Quantum optics, Qubits, Superconducting devices, Photonic devices

## Abstract

We investigate a hybrid device allowing a photon–phonon coupling of a transmission line radiation (TLR) and a nanoeletromechanical system (NEMS), mediated by a superconducting qubit population imbalance. We demonstrate the derivation of an effective Hamiltonian for the strongly dispersive regime for this system. The qubit works as a quantum switch, allowing a conditioned transfer of excitations between the TLR and NEMS. We show that this regime allows the system to be employed for signal processing and force estimation. Additionally, we explore the ability of the quantum switch to generate non-classical states.

## Introduction

The emerging field of hybrid cavity quantum electrodynamics (CQED) with nanoelectromechanical systems (NEMS) has great potential for quantum signal processing applications^1–4^. The interaction between light and matter, confined in a resonant cavity, allows precise control and manipulation of quantum states for certain conditions. NEMS refer to devices at the nanometre scale that exhibit mechanical excitations and are highly sensitive to external potential stimuli. By combining these two systems, a hybrid device is formed that allows for a better architecture for signal detection and estimation, as well as for signal processing^5^.

The experimental advances on the implementation of CQED-NEMS devices has allowed greater sensitivity, low noise, and potential scalability^6^. The ability to achieve strong couplings allows for efficient transfer of signal information, resulting in greater sensitivity compared to traditional detection schemes^7–13^. In addition, the inherent quantum properties of the hybrid system offer unique opportunities for quantum-enhanced signal processing and estimation, including quantum entanglement, quantum measurement and quantum sensing techniques.

In this paper, we investigate a detection scheme mediated by a photon–phonon coupled by a Cooper pair within a platform comprising a CQED-NEMS device. Our primary focus is on the investigation of the relationship between signal processing and force estimation within this unique circuit, through an effective Hamiltonian tailored specifically for the strongly dispersive regime. We show that the photon–phonon interaction mediated by a cooper pair in a dispersive regime can be effectively written as a beam-splitter conditioned on the state of a quantum bit (qubit), which allows for signal enhancement and enables the generation of entangled states via a quantum switch.

## Physical system

**Figure 1:**
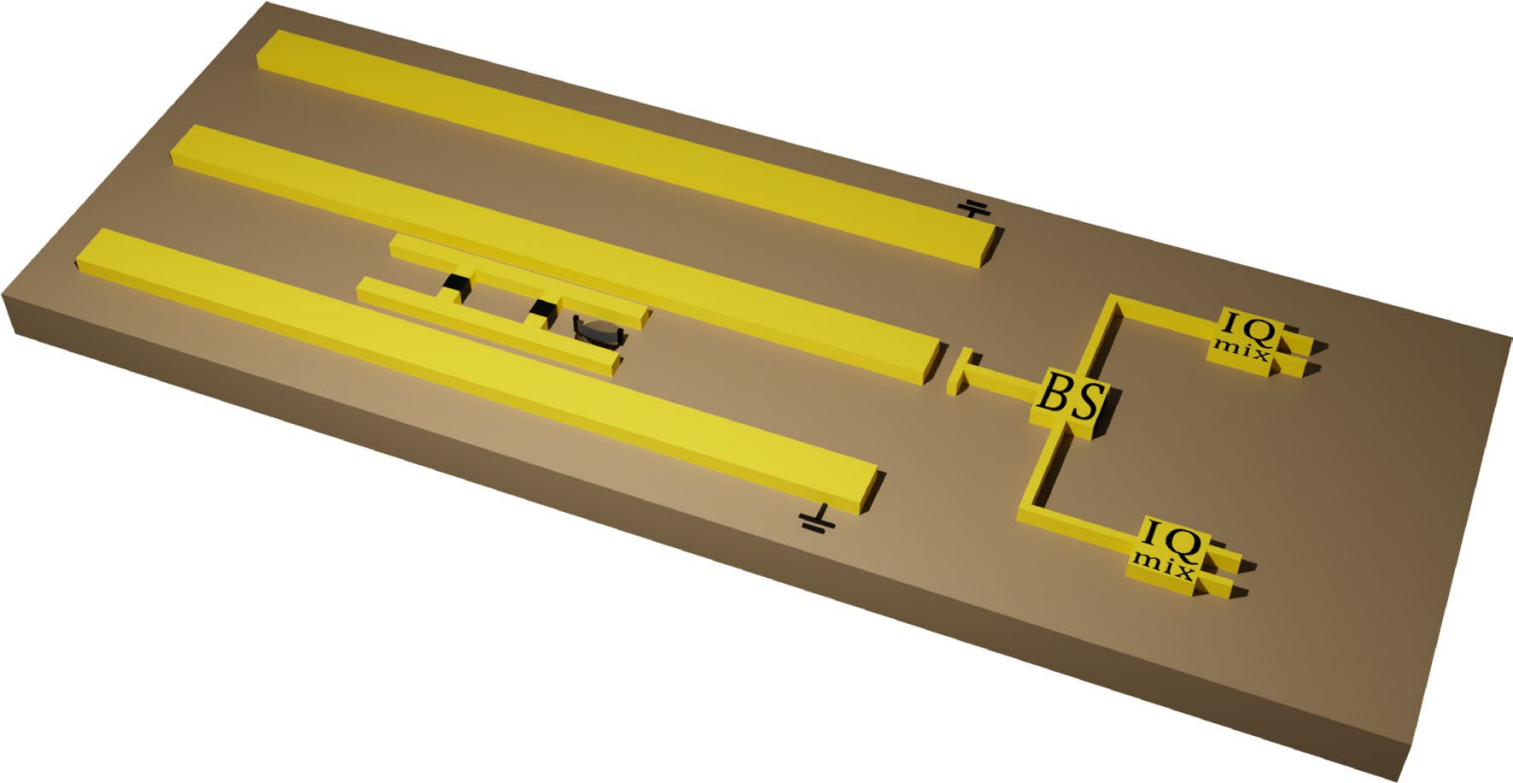
Schematic of a hybrid quantum circuit with IQ mixer measurements:The circuit schematized on a platform contains an electromagnetic shield with two grounded lines, and between them is a quantum hybrid circuit composed of the capacitive coupling between a TLR and a suspended NEMS mediated by a qubit superconducting^2,5^. The TLR also has a channel capacitively coupled to its output that connects a beam splitter (BS) with two linear detectors for IQ mix measurements^16,17^.

**Figure 2:**
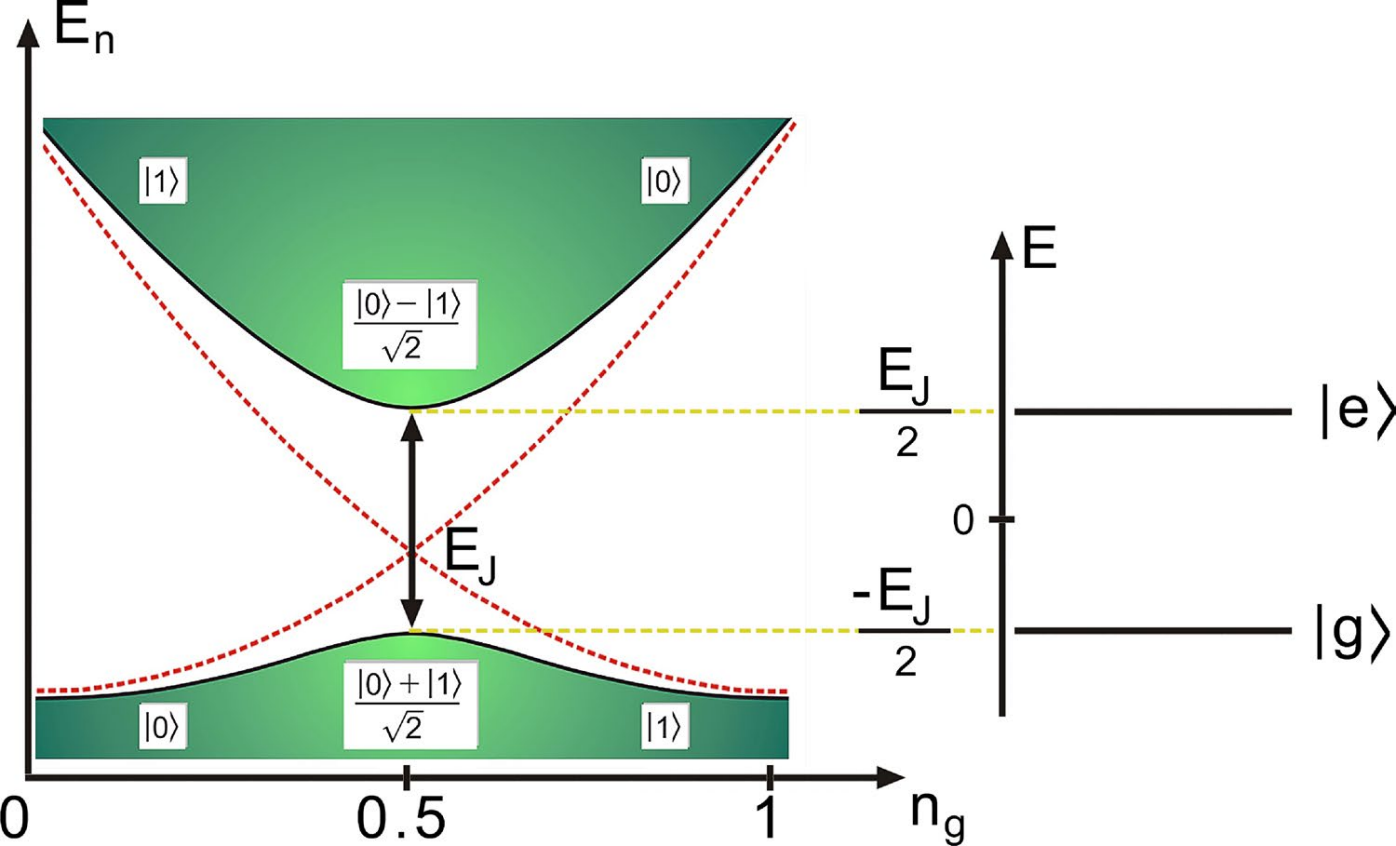
Representation of qubit energy levels as a function of gate charge, $n_{g}$, can be rewritten as a two-level quantum system with transition energy $E_{J}$ and hybrid coupling energy, $\frac{E_{0}-E_{1}}{2}$, (light-matter-mechanical oscillator).

The circuit under study consists of a transmission line resonator (TLR) capacitively coupled to a NEMS through a charge qubit, containing a single Cooper pair box (CPB) made of a superconducting island connected to a large reservoir through two Josephson tunnel junctions with Josephson energy and capacitance $E_{J}$ and $C_{J}$, respectively. The Cooper pair box is capacitively coupled to the TLR and the NEMS with gate capacitances $C_{t}$ and $C_{n}$, respectively (see Fig. 1). In the Cooper-pairs number basis, the system Hamiltonian can be written as^14,15^

1 $$\begin{aligned} \hat{H} = \sum _{n} 4E_{c}({n}-n_{g})^{2} \left| n\right\rangle \left\langle n\right| - \frac{E_{J}}{2}\left( \left| n\right\rangle \left\langle n+1\right| +\left| n+1\right\rangle \left\langle n\right| \right) , \end{aligned}$$

where $E_{c}=e/2C_{\sum }$ is the single electron charging energy (*e* is the electronic charge), $C_{\sum }=C_{J}+C_{t}+C_{n} \approx C_{J}+C_{t}$ is its total capacitance^2^, *n* represents the expected Cooper pair number on the island, and $n_{g}$ is the gate charge applied to it. Following the description, for ${n}=0,1$, (associated with the accumulation of a single Cooper pair on the island—a current assumption when the superconducting system is at the point of charge degeneracy for states $\{|0\rangle , |1\rangle \}$, where any transition to states of higher excitation is suppressed^2^, see Fig. 2), the Hamiltonian (1) is rewritten as

2 $$\begin{aligned} \hat{H}= & {} E_{0} \left| 0\right\rangle \left\langle 0\right| + E_{1} \left| 1\right\rangle \left\langle 1\right| - \frac{E_{J}}{2}\left( \left| 0\right\rangle \left\langle 1\right| +\left| 1\right\rangle \left\langle 0\right| \right) , \end{aligned}$$

where $$E_{0}=4E_{c}n_{g}^{2}$$ and $$E_{1}=4E_{c}( 1 - n_{g}^{2}) ^{2}.$$

By changing the base of the CPB to

$$\begin{aligned} \left| g\right\rangle= & {} \frac{\left| 0\right\rangle + \left| 1\right\rangle }{\sqrt{2}}, \end{aligned}$$

and

$$\begin{aligned} \left| e\right\rangle= & {} \frac{\left| 0\right\rangle - \left| 1\right\rangle }{\sqrt{2}}, \end{aligned}$$

we have the Hamiltonian (2) represented:

3 $$\begin{aligned} \hat{H}= & {} \frac{\left( E_{0} - E_{1} \right) }{2} \hat{\sigma }_{x} + \frac{E_{J}}{2}\hat{\sigma }_{z}, \end{aligned}$$

where $E_{1} - E_{0}=4E_{c}\left( 1-2n_{g} \right)$, $\hat{\sigma }_{x}=\left| g\right\rangle \left\langle e\right| +\left| e\right\rangle \left\langle g\right|$ and $\hat{\sigma }_{z}=\left| g\right\rangle \left\langle g\right| -\left| e\right\rangle \left\langle e\right|$. From now on we call it the qubit base (See Fig. 2).

Now we proceed to the proper treatment for the interaction between qubit-TLR and qubit-NEMS by extending the quantization to the gate charge $n_{g}\rightarrow n_{0} + \delta \hat{n}_{t}+\delta \hat{n}_{n}$, where^2,6, 18, 19^

$$\begin{aligned} n_{0}= & {} \frac{C_{t}V_{0}}{2e}, \end{aligned}$$

is the the bias gate composed of a DC field $V_{0}$ in TLR, the

$$\begin{aligned} \delta \hat{n}_{t} = \frac{C_{t}}{2e}\hat{V}(t) = C_{t}\sqrt{\frac{\hbar \omega _{t}}{Lc}}\frac{\left( \hat{a}e^{-i\omega _{t}t}+\hat{a}^{\dag }e^{i\omega _{t}t}\right) }{2e}, \end{aligned}$$

is the induced charge due to the TLR^14^, being $\hat{V}(t)=\sqrt{\frac{\hbar \omega _{t}}{Lc}}\left( \hat{a}e^{-i\omega _{t}t}+\hat{a}^{\dag }e^{i\omega _{t}t}\right)$, the voltage at the center of the TLR of length *L*, and capacitance density *c*. Here, $\omega _{t}$ and $\hat{a}$($\hat{a}^{\dag }$) are the frequency and annihilation (creation) operator of the TLR, respectively, and

$$\begin{aligned} \delta \hat{n}_{n} = \frac{V_{n}}{2e}C_{n}(\hat{x}(t)), \end{aligned}$$

represents the NEMS flexural mode shift, with $V_{n}$ is the potential difference that can be applied to the NEMS, the capacitance formed between the NEMS and the qubit can be expressed and approximated as

$$\begin{aligned} C_{n} \equiv C_{n}(\hat{x}) = \frac{\varepsilon_{o}A}{d-\hat{x} } \approx \frac{\varepsilon_{o}A}{d} \left\{ 1 + \frac{\hat{x}}{d} + \mathscr {O}(2) + \cdots \right\} , \end{aligned}$$

with $\varepsilon_{o}$ is Vacuum permittivity, *A* is the rara of the lateral longitudinal section of the NEMS, and *d* is the equilibrium distance between the NEMS and the qubit. Therefore, using only the first-order term, we obtain^20^

$$\begin{aligned} \delta \hat{n}_{n} \approx \frac{dC_{n}}{dx}\sqrt{\frac{\hbar }{2 m \omega _{n}}}V_{n} \frac{\left( \hat{b}e^{-i\omega _{n}t}+\hat{b}^{\dag }e^{i\omega _{n}t}\right) }{2e}, \end{aligned}$$

where $\hat{x}=\sqrt{\frac{\hbar }{2 m \omega _{n}}}\left( \hat{b}e^{-i\omega _{n}t}+\hat{b}^{\dag }e^{i\omega _{n}t}\right)$ representing the quantised bending mode of NEMS (equivalent to a quantum harmonic oscillator), with $\omega _{n}$, *m*, $\frac{dC_{n}}{dx}\approx \frac{\varepsilon_{o}A}{d^{2}}$ and $\hat{b}$($\hat{b}^{\dag }$) being the frequency, mass, first order coefficient of the capacitance expansion $C_{n} \equiv C_{n}(\hat{x})$, and the NEMS annihilation (creation) operator, respectively. Now, to better represent Hamiltonian (3), we rewrite

4 $$\begin{aligned} \hat{H}= & {} -2E_{c}\left[ 1-2 \hat{n}_{g} \right] \hat{\sigma }_{x} +\frac{E_{J}}{2}\hat{\sigma }_{z}\nonumber \\= & {} - 2E_{c}\left[ 1 - 2 \hat{n}_{o} - 2 \delta \hat{n}_{t} - 2\delta \hat{n}_{n} \right] \hat{\sigma }_{x} +\frac{E_{J}}{2}\hat{\sigma }_{z}, \end{aligned}$$

with $$n_{o}=0.5,$$

5 $$\begin{aligned} \hat{H}= & {} \frac{E_{J}}{2}\hat{\sigma }_{z} + 4E_{c}\left[ \delta \hat{n}_{t} + \delta \hat{n}_{n} \right] \hat{\sigma }_{x} \nonumber \\= & {} \frac{\hbar \omega _{q}}{2}\hat{\sigma }_{z} + \hbar \Omega \left( \hat{a}e^{-i\omega _{t}t}+\hat{a}^{\dag }e^{i\omega _{t}t}\right) \hat{\sigma }_{x} + \hbar \lambda \left( \hat{b}e^{-i\omega _{n}t}+\hat{b}^{\dag }e^{i\omega _{n}t}\right) \hat{\sigma }_{x}, \nonumber \\ \end{aligned}$$

where, $\hbar \Omega = \frac{2E_{c}C_{t}}{e}\sqrt{\frac{\hbar \omega _{t}}{Lc}}$, $\hbar \lambda = \frac{2E_{c}}{e}\sqrt{\frac{\hbar }{2m\omega _{n}}}\frac{dC_{N}}{dx}V_{N}$, and $\hbar \omega _{q}=E_{J}$. Note that $\lambda$ is manipulated by $V_{N}$, and $\omega _{q}$ is manipulated by classical flux $\Phi$ applied to the qubit loop^6^. In the interaction picture obtained by the unitary transformation $\hat{U}_{t}=e^{-i\hat{H}_{o}t/\hbar }$, where $\hat{H}_{o}= \hbar \omega _{q}\hat{\sigma }_{z}/2$, corresponding to the qubit free evolution, the Hamiltonian becomes (with $\hat{\sigma }_{+}=\left| e\right\rangle \left\langle g\right|$ and $\hat{\sigma }_{-}=\left| g\right\rangle \left\langle e\right|$)

6 $$\begin{aligned} \hat{H}^{I}= & {} \hbar \Omega \left( \hat{a}e^{-i\omega _{t}t}+\hat{a}^{\dag }e^{i\omega _{t}t}\right) \left( \hat{\sigma }_{+}e^{i\omega _{q}t} + \hat{\sigma }_{-}e^{-i\omega _{q}t} \right) + \hbar \lambda \left( \hat{b}e^{-i\omega _{n}t}+\hat{b}^{\dag }e^{i\omega _{n}t}\right) \left( \hat{\sigma }_{+}e^{i\omega _{q}t} + \hat{\sigma }_{-}e^{-i\omega _{q}t} \right) . \end{aligned}$$

The overall dynamics could be analyzed from this Hamiltonian, however, we can keep only the more relevant terms in the regime in which $\omega _{t}+\omega _{q} \gg |\omega _{t}-\omega _{q}|$, $\omega _{n}+\omega _{q} \gg |\omega _{n}-\omega _{q}|$, the rotating wave approximation can be assumed, and all the remaining oscillating terms are neglected, resulting for the complete Hamiltonian

7 $$\begin{aligned} \hat{H}= & {} \hbar \frac{\omega _{q}}{2}\hat{\sigma }_{z} + \hbar \omega _{t}\hat{a}^{\dag }\hat{a} + \hbar \omega _{n}\hat{b}^{\dag }\hat{b} + \hbar \Omega \left( \hat{\sigma }_{+}\hat{a} + \hat{\sigma }_{-}\hat{a}^{\dag } \right) + \hbar \lambda \left( \hat{\sigma }_{+}\hat{b} + \hat{\sigma }_{-}\hat{b}^{\dag } \right). \end{aligned}$$

Remark that the counter-rotating and oscillatory terms would only introduce perturbative modulations on the mean contribution of the rotating terms, with no further consequence for the present results.

Finally, we assume a strongly dispersive regime^21^ (with $\left| \frac{\lambda ^{2}}{\omega _{n}-\omega _{q}}\right| \approx \left| \frac{\Omega ^{2}}{\omega _{t}-\omega _{q}}\right| >\Gamma$, where $\Gamma =max\left[ \gamma ,\kappa _{t},\kappa _{t}\right]$ being $\gamma$, $\kappa _{t}$, and $\kappa _{n}$ the dissipation rates of the qubit, TLR, and the NEMS, respectively. This assumption is in agreement with actual experimental possibilities according to references^21–24^). To simplify, we can take the Baker-Campbell-Hausdorff expansion $e^{\hat{A}}\hat{H}e^{-\hat{A}} \approx \hat{H} + \left[ \hat{A},\hat{H} \right]$ with $\hat{A} = \xi _{1} \left( \hat{\sigma }_{+}\hat{a} - \hat{\sigma }_{-}\hat{a}^{\dag } \right) + \xi _{2} \left( \hat{\sigma }_{+}\hat{b} - \hat{\sigma }_{-}\hat{b}^{\dag } \right)$, for $\xi _{1}=\frac{\Omega }{\delta _{t}}$ and $\xi _{2}=\frac{\lambda }{\delta _{n}}$, with $\delta _{n}=\omega _{n}-\omega _{q}$ and $\delta _{t}=\omega _{t}-\omega _{q}$, to write $\hat{H}_{eff}=e^{\hat{A}}\hat{H}e^{-\hat{A}}$, resulting in the effective Hamiltonian

8 $$\begin{aligned} \hat{H}_{eff}\approx & {} \hbar \omega _{t}\hat{a}^{\dag }\hat{a} + \hbar \omega _{n}\hat{b}^{\dag }\hat{b} + \hbar \chi _{t}\hat{a}^{\dag }\hat{a}\hat{\sigma }_{z} + \hbar \chi _{n}\hat{b}^{\dag }\hat{b}\hat{\sigma }_{z} + \hbar \chi \hat{\sigma }_{z} \left( \hat{a}\hat{b}^{\dag }+\hat{a}^{\dag }\hat{b} \right) , \end{aligned}$$

where $$\chi _{t}=2\Omega \xi _{1}$$, $$\chi _{n}=2\lambda \xi _{2}$$ and $$\chi = \lambda \xi _{1} +\Omega \xi _{2}$$.

### IQ mixer measurement scheme

In this Section we introduce the detection procedure, already explored in several other references^16,17^. By adding a drive with amplitude $\varepsilon$ on the NEMS

$$\begin{aligned} \hat{H}_{d}= & {} \hbar \varepsilon\left( \hat{b}^{\dag } + \hat{b} \right) , \end{aligned}$$

we can rewrite the effective Hamiltonian in interaction representation

9 $$\begin{aligned} \hat{{H}}^{I}_{eff}\approx & {} \hbar \chi \hat{\sigma }_{z} \left( \hat{a}\hat{b}^{\dag }+\hat{a}^{\dag }\hat{b} \right) + \hbar \varepsilon\left( \hat{b} + \hat{b}^{\dag } \right) . \end{aligned}$$

This Hamiltonian shows a beam-splitter-like interaction for phonon and photons, mediated by the qubit state population imbalance, plus the driving on the NEMS. This conditioned interaction allows the qubit to work as a switch for the NEMS and cavity excitations interchange. There are previous examples in the literature illustrating the use of a quantum switch^18^ architecture to generate non-classical states of microwave radiation and to establish entanglement between the modes of the resonator and the degrees of freedom of the qubit. Here, the term “qubit” refers specifically to the qubit used for the operation of the quantum switch.

Let us consider the generation and transfer of superposition states and the entanglement between the resonators and the qubit. According to the Hamiltonian (9), given the initial state in the form

10 $$\begin{aligned} \left| \psi _{0}\right\rangle= & {} \left[ \sin (\theta ) \left| g \right\rangle + \cos (\theta ) \left| e \right\rangle \right] \otimes \left| 0,0 \right\rangle \nonumber \\= & {} \sin (\theta ) \left| g,0_{g},0_{g} \right\rangle + \cos (\theta ) \left| e,0_{e},0_{e} \right\rangle , \end{aligned}$$

where $\left| g \right\rangle$ is the ground state, and $\left| e \right\rangle$ is the excited state of qubit, the evolved state is written as

11 $$\begin{aligned} \left| \psi _{t}\right\rangle= & {} e^{\frac{-i {\hat{H}}^{I}_{eff} t}{\hbar }} \left| \psi _{0}\right\rangle \nonumber \\= & {} \sin (\theta ) \left| g,\alpha _{g},\beta _{g} \right\rangle + \cos (\theta ) \left| e,\alpha _{e},\beta _{e} \right\rangle , \end{aligned}$$

where $\left| \alpha _{\sigma } \right\rangle$ and $\left| \beta _{\sigma } \right\rangle$ are coherent states of the TLR and NEMS, respectively, for $\sigma =g,e$.

Therefore, with $\left\langle g |\hat{\sigma }_{z}| g \right\rangle =-1$, $\left\langle e |\hat{\sigma }_{z}|e\right\rangle =1$, $\left\langle \hat{a} \right\rangle _{\sigma }=\alpha _{\sigma }$, and $\left\langle \hat{b} \right\rangle _{\sigma }=\beta _{\sigma }$ we have the following set of equations

12a $$\begin{aligned} \dot{\alpha }_{g}= & {} + i\chi \beta _{g}, \end{aligned}$$

12b $$\begin{aligned} \dot{\beta }_{g}= & {} + i\chi \alpha _{g} - i\varepsilon, \end{aligned}$$

12c $$\begin{aligned} \dot{\alpha }_{e}= & {} - i\chi \beta _{e}, \end{aligned}$$

12d $$\begin{aligned} \dot{\beta }_{e}= & {} - i\chi \alpha _{e} - i\varepsilon, \end{aligned}$$

whose solutions are

13a $$\begin{aligned} \alpha _{g}= & {} \frac{\varepsilon}{\chi }\left[ 1-\cos (\chi t) \right] , \end{aligned}$$

13b $$\begin{aligned} \beta _{g}= & {} -i \frac{\varepsilon}{\chi } \sin (\chi t), \end{aligned}$$

13c $$\begin{aligned} \alpha _{e}= & {} \frac{\varepsilon}{\chi }\left[ \cos (\chi t) - 1 \right] , \end{aligned}$$

13d $$\begin{aligned} \beta _{e}= & {} - \frac{i\varepsilon}{\chi } \sin (\chi t). \end{aligned}$$

The output of the TLR is directed to a microwave beam-splitter (BS), as can be seen in Fig. 1. The two outputs then pass through separate IQ mixers^16,17^. The four output currents from two IQ mixers can be correlated in various ways. Our particular used observable is the microwave field quadrature

14 $$\begin{aligned} \hat{X}_{\phi }= & {} \frac{\hat{a}e^{-i\phi } + \hat{a}^{\dag } e^{i \phi } }{2}, \end{aligned}$$

which gives direct access to all the normally ordered moments of the TLR field at the instant of measurement. The cross-correlations are calculated after the entire field has been detected and the TLR has returned to the vacuum state, ready for repetition of the same process. This results in the value of the quadrature signal measured at the TLR as

15 $$\begin{aligned} \left\langle \hat{X}_{\phi } \right\rangle = \frac{\varepsilon}{\chi }\left[ 1- \cos (\chi t) \right] \left[ \sin (\theta ) - \cos (\theta )\right] \cos (\phi ). \end{aligned}$$

**Figure 3:**
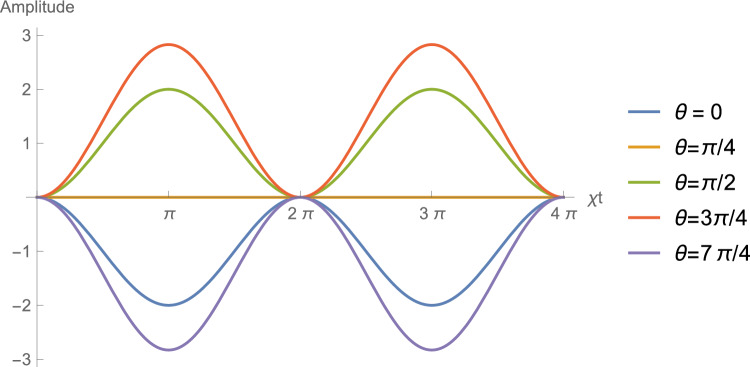
Rate quadrature signal measured at the TLR by input sinal in the NEMS, $\chi \left\langle \hat{X}_{\phi =0} \right\rangle /\varepsilon$ in function of $\chi t$ to different $\theta$, Eq. (15).

With a simple differential analysis of the critical points, we can qualify the signal processing through the state of the quantum switch, where we have the maximum intensities at $\theta _{max}=(8n+3)\pi /4$, minimum at $\theta _{min}=(8n+7)\pi /4$, and no processing at $\theta _{0}=(4n+1)\pi /4$, for $n=...,-3,-2,-1,0,1,2,3,...$, all with the best detected quadrature $\hat{X}_{\phi =0}=\hat{X}$. The dynamical behavior is periodic, as shown in Fig. 3.

### Fisher information associated with the quadrature measurement

The positive operator associated with a quadrature measurement is given by

16 $$\begin{aligned} {\hat{\Pi }}(x)= \vert x_{\phi }\rangle \langle x_{\phi } \vert . \end{aligned}$$

Notice that the results obtained in the measurement of a given quadrature are parameterized by a continuous variable *x*. Here is the value of $\phi$ that establishes the quadrature to be measured. The probability density of finding the value of a quadrature in $\phi$ with the value *x* is given by

17 $$\begin{aligned} \mathscr {P}_{\phi }(\varepsilon\vert x) = \langle x_{\phi } \vert \hat{\rho }_A(t) \vert x_{\phi } \rangle , \end{aligned}$$

where the state $\vert x_\phi \rangle$ is defined as being the eigenstate of the quadrature $\hat{X}_{\phi }$, with eigenvalue $x_\phi$. The probability density $\mathscr {P}(\varepsilon\vert x)$ may be described as

18 $$\begin{aligned} \mathscr {P}_{\phi }(\varepsilon\vert x)= & {} \sum _{\sigma =g,e} p_{\theta } \langle x_{\phi }\vert \alpha _{\sigma } \rangle \langle \alpha _{\sigma } \vert x_{\phi } \rangle \nonumber \\= & {} \sin ^{2}(\theta ) \left| \psi _{\alpha _{g}} (x_{\phi }) \right| ^{2} + \cos ^{2}(\theta ) \left| \psi _{\alpha _{e}} (x_{\phi }) \right| ^{2}. \end{aligned}$$

However, $\psi _{\alpha _{\sigma }} \equiv \psi _{\alpha _{\sigma }} (x_{\phi }) = \left\langle x_{\phi } \vert \alpha _{\sigma } \right\rangle$ can be calculated by identifying that $\hat{a}\left| \alpha _{\sigma }\right\rangle = \alpha _{\sigma } \left| \alpha _{\sigma }\right\rangle$, and so $\left\langle x_\phi \right| \hat{a}\left| \alpha _{\sigma }\right\rangle = \alpha _{\sigma } \left\langle x_{\phi } \vert \alpha _{\sigma }\right\rangle$. Therefore,

$$\begin{aligned} \left\langle x_{\phi } \right| \left\{ \hat{X}_{\phi } + i\hat{X}_{\phi +\frac{\pi }{2}} \right\} \left| \alpha _{\sigma }\right\rangle e^{i\phi } = \alpha _{\sigma } \left\langle x_{\phi } \vert \alpha _{\sigma }\right\rangle . \end{aligned}$$

This is the same as the differential equation

19 $$\begin{aligned} 2 x_{\phi }\psi _{\alpha _{\sigma }}+\frac{\partial }{\partial x_{\phi }}\psi _{\alpha _{\sigma }}= & {} 2\alpha _{\sigma } e^{-i\phi } \psi _{\alpha _{\sigma }}, \end{aligned}$$

with the normalization

$$\begin{aligned} \int _{-\infty }^{\infty } \vert \psi _{\alpha _{\sigma }} \vert ^{2} dx_{\phi }= & {} 1. \end{aligned}$$

**Figure 4:**
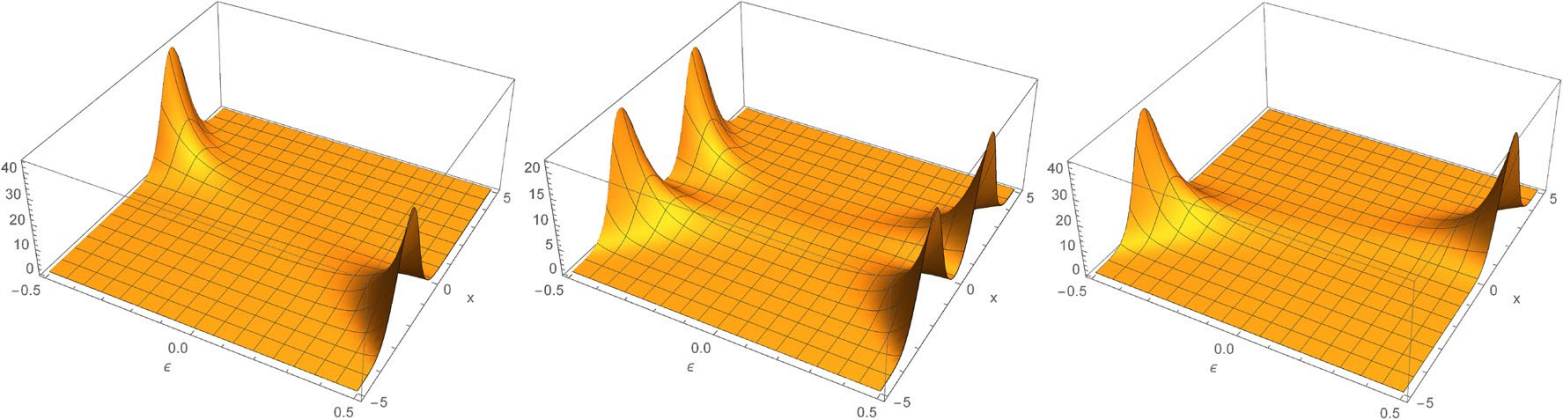
$\mathscr {P}_{\phi }(\varepsilon\vert x)$, for $\theta =0$ (left), (and $\theta =\pi /4$ $3\pi /4$, $7\pi /4$) (central), and $\theta =\pi /2$ (right), with $\phi = 0$ and $\chi t= \pi$ in all.

Equation (19) has the solution

20 $$\begin{aligned} \psi _{\alpha _{\sigma }}(x_{\phi })= & {} \left( \frac{2}{\pi } \right) ^{1/4} e^{- |\alpha _{\sigma }|^{2}} e^{- x_{\phi }^{2} + 2 \alpha _{\sigma }e^{-i\phi } x_{\phi } }, \end{aligned}$$

resulting in a mixture of two Gaussian distributions of the form

21 $$\begin{aligned} \mathscr {P}_{\phi }(\varepsilon\vert x)= & {} \sin ^{2}(\theta )\left( \frac{2}{\pi } \right) ^{1/2} e^{- x_{\phi }^{2} + 2 \left( \alpha _{g}e^{-i\phi } + \alpha _{g}^{*}e^{i\phi } \right) x_{\phi } } + \cos ^{2}(\theta )\left( \frac{2}{\pi } \right) ^{1/2} e^{- x_{\phi }^{2} + 2 \left( \alpha _{e}e^{-i\phi } + \alpha _{e}^{*}e^{i\phi } \right) x_{\phi } }, \end{aligned}$$

characterized by the driving force $\varepsilon$. The profile of $\mathscr {P}_{\phi }(\varepsilon\vert x)$ shows a distinct change of symmetry as $\theta$ is varied, as shown in Fig. 4, for $\phi =0$ and $\chi t=\pi$.

**Figure 5:**
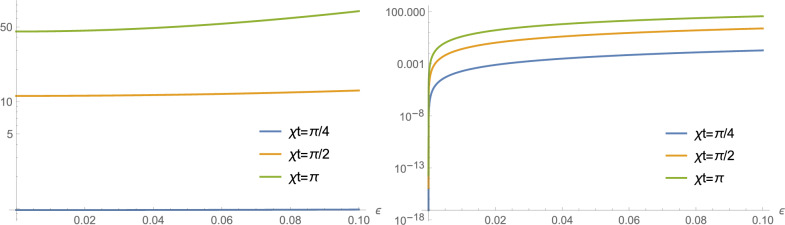
LogPlot of $F(\varepsilon,\theta ,\phi )$ in function of $\varepsilon$, for $\theta =0,\pi /2$ (left), and $\theta =\pi /4,3\pi /4,7\pi /4$ (right), with $\phi = 0$ in all. This shows that, regardless of the intensity of the signal and depending on the behaviour of $\mathscr {P}_{\phi }(\varepsilon\vert x)$, the superposition of the qubit state can directly influence the estimate of $\varepsilon$, at different times with a sudden or gradual increase in LogPlot of $F(\varepsilon,\theta ,\phi )$ in relation to $\varepsilon$.

The Fisher information associated with this measurement can be employed to infer the optimal parameters for inference of $\varepsilon$. It is calculated with the expression

22 $$\begin{aligned} F(\varepsilon;\theta ,\phi ) = \int _{-\infty }^{+\infty } \frac{dx_{\phi }}{\mathscr {P}_{\phi }(\varepsilon\vert x)}\left( \frac{\partial \mathscr {P}_{\phi }(\varepsilon\vert x)}{\partial \varepsilon}\right) ^2. \end{aligned}$$

The numerical calculation of equation (22) in an integration in the interval between $-30\le x_{\phi =0} \le 30$ results in the graph in Fig. 5 with plots for different instants, showing good estimates of the force $\varepsilon$ even for the situation with null average signal, as described in Fig. 3. Remark that depending on the behaviour of $\mathscr {P}_{\phi }(\varepsilon\vert x)$, illustrated in Fig. 4, the superposition of the qubit state can directly influence the estimate of $\varepsilon$, at different times with a sudden or gradual increase in the LogPlot of $F(\varepsilon,\theta ,\phi )$ in relation to $\varepsilon$.

### A quantum switch protocol

Given the optimal force estimation for $\theta =3\pi /4$, we can extend the proposal to use the quantum switch to generate nonclassical states. For example, one could employ it to generate operations on the“qubit-like” states^25^ encoded in superposition states of the TLR field^26,27^ as

$$\begin{aligned} \left| 0 \right\rangle _{L}^{\alpha }= & {} \frac{\left| \alpha \right\rangle - \left| -\alpha \right\rangle }{\sqrt{2}}, \end{aligned}$$

and

$$\begin{aligned} \left| 1 \right\rangle _{L}^{\alpha }= & {} \frac{\left| \alpha \right\rangle + \left| -\alpha \right\rangle }{\sqrt{2}}. \end{aligned}$$

Here we generate the Hadamard operation, simply by returning the base of the qubit to base $\left| 0\right\rangle$ and $\left| 1\right\rangle$ in state (11), (with $\alpha _{g}=-\alpha _{e}=\alpha$ and $\beta _{g}=\beta _{e}=\beta$)

23 $$\begin{aligned} \left| \psi _{t}\right\rangle= & {} \sin (3\pi /4) \left| g,\alpha ,\beta \right\rangle + \cos (3\pi /4) \left| e,- \alpha ,\beta \right\rangle \nonumber \\= & {} \frac{\left[ \left( \left| 0 \right\rangle + \left| 1\right\rangle \right) \left| \alpha \right\rangle - \left( \left| 0 \right\rangle - \left| 1\right\rangle \right) \left| -\alpha \right\rangle \right] }{2} \left| \beta \right\rangle \nonumber \\= & {} \frac{\left[ \left| 0 \right\rangle \left| 0 \right\rangle _{L}^{\alpha } + \left| 1 \right\rangle \left| 1 \right\rangle _{L}^{\alpha } \right] }{\sqrt{2} } \left| \beta \right\rangle . \end{aligned}$$

**Figure 6:**
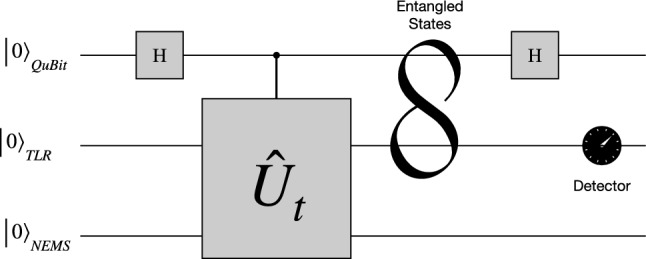
Schematic diagram of a Logic operaction.

Note that applying a new pulse to the NEMS can generate a CNOT logic gate, see Fig. 6. Other types of states can also be generated, such as GHZ^28^, and GKP states^29^.

## Concluding remarks

In view of this, we conclude that the present hybrid circuit in the strongly dispersive regime presents a good means of processing the photon–phononic signal and an excellent detector through the quadrature measurement scheme using linear detectors.

The exploration of quantum switches and their role in entanglement phenomena are of remarkable relevance for quantum information processing^30,31^. The examples discussed in this context, particularly focusing on the force estimation, the creation of nonclassical states of microwave radiation, and the establishment of entanglement between NEMS, TLR and qubit, highlight the potential of quantum switches in manipulating quantum states for various applications.

The viability of implementing experimentally the present proposal is very high, considering that nearly a decade ago, in a similar circuit architecture^6^, it was possible to achieve values where $\Omega =120\,{\text{MHz}}$, $\lambda =1.35\,{\text{MHz}}$, $\delta _{n}= - 0.73\,{\text{GHz}}$, $\delta _{T}=0.74\,{\text{GHz}}$, and that it has recently been shown that the devices involved can have dissipation rates of the order of a few *kHz*^32–35^. Previously, the implementation of the mixed IQ measurement for TLR was experimentally carried out in reference^36,37^.

The ability to exploit the quantum switch architecture for tasks such as coherent state transfer and the generation of entanglement provides a promising avenue for advancing quantum communication and computation technologies. These findings contribute to the growing body of knowledge in the field of quantum information science, offering insights into the controlled manipulation of quantum states at the quantum hybrid circuit level.

## Data Availability

All data generated or analysed during this study are included in this published article.
